# Homelessness and health-related outcomes in the Republic of Ireland: a systematic review, meta-analysis and evidence map

**DOI:** 10.1007/s10389-023-01934-0

**Published:** 2023-06-01

**Authors:** Carolyn Ingram, Conor Buggy, Darin Elabbasy, Carla Perrotta

**Affiliations:** 1 School of Public Health, Physiotherapy, and Sports Science, University College Dublin, D04 V1W8 Dublin, Ireland; 2 Centre for Safety and Health at Work, University College Dublin, D04 V1W8 Dublin, Ireland

**Keywords:** Homelessness, Republic of Ireland, Health Disparities, Evidence Map, Substance Use, Mental Health

## Abstract

**Aim:**

To map existing research on homelessness and health in the Republic of Ireland, and to synthesize the evidence on housing-related disparities in health.

**Methods:**

Peer-reviewed articles and conference abstracts published in English between 2012–2022 were retrieved from 11 bibliographic databases if they contained empirical data on homelessness and health in Ireland, and – in a subsequent screening stage – at least one measure of health disparity between the homeless and general populations. Reviewers extracted relative risks (RR), 95% confidence intervals (CI), and calculated pooled RR of comparable health disparities using pairwise random-effects meta-analyses.

**Results:**

One hundred four articles contained empirical data on the health of homeless individuals residing in Ireland, addressing primarily substance use, addiction and mental health. Homelessness was associated with increased risk of illicit drug use (RR 7.33 [95% CI 4.2, 12.9]), reduced access to a general practitioner (GP) (RR 0.73 [CI 95% 0.71, 0.75]), frequent emergency department (ED) presentation (pooled RR 27.8 [95% CI 4.1, 189.8]), repeat presentation for self-harm (pooled RR 1.6 [95% CI 1.2, 2.0]) and premature departure from hospital (pooled RR 2.65 [95% CI 1.27, 5.53]).

**Conclusions:**

Homelessness in Ireland is associated with reduced access to primary care and overreliance on acute care. Chronic conditions amongst homeless individuals are understudied.

**Supplementary Information:**

The online version contains supplementary material available at 10.1007/s10389-023-01934-0.

## Introduction

Homelessness levels continue to rise in most parts of Europe with children and adolescents, migrants, ethnic minorities, women, and families at increasing risk (European Commission 2022). The Republic of Ireland is unique in the severity of recent national spikes in homelessness (Allen et al. 2020). According to official administrative data, the number of people staying in overnight emergency accommodation more than doubled in Ireland since 2015, reaching a record high of 7917 adults and 3480 children in October 2022 (Department of Housing, Local Government and Heritage 2022). The number of families who are homeless increased by 300% (Long et al. 2019). These figures do not account for ‘hidden homelessness’ (Allen et al. 2020); an estimated 290,000 adults slept in their cars, in squats, on the floors or sofas of family and friends, or in unsafe accommodation in 2022 (~6% of Irish population) (RED C and Simon Community 2022). Such trends come at high social and personal cost. Individuals and families experiencing homelessness face discrimination, isolation, barriers to accessing public services, and in turn, health disparities (European Commission 2022).

Health disparities are chains of events signified by differences in environment, access to, utilization of, and quality of care, overall health status, or a particular health outcome (Carter-Pokras and Baquet 2002). These differences encompass health inequalities (i.e. variation in health status or outcomes) and inequities (i.e. variation in the distribution or allocation of resources), and adversely affect groups of people who experience greater social or economic obstacles to health based on race or ethnicity, religion, socioeconomic status (SES), gender, age, disability, geographic location, sexual orientation, or other characteristics historically linked to discrimination or exclusion (Klein and Huang 2010). The mechanisms leading to disparities in health are complex. Structural, place-based, and individual factors such as concentrated poverty, proximity to environmental hazards, access to healthcare, stress and lifestyle risk factors intersect and contribute to adverse health outcomes in marginalized populations (Fazel et al. 2014; Ingram et al. 2021). Social exclusion, by determining access to education and employment opportunities, impedes economic opportunity. SES, in turn, shapes material circumstances and health risk behaviours contributing to overall health and well-being (World Health Organization 2010). Woodward and Kawachi (2000) argue that resulting health inequalities are unjust, avoidable, expensive and detrimental to all members of society (Woodward and Kawachi 2000).

Health disparities amongst individuals experiencing homelessness have been widely documented. Homeless individuals in high-income countries have higher rates of suicide and unintentional injuries and an increased prevalence of a range of infectious diseases, mental disorders and substance misuse (Fazel et al. 2014). High rates of age-related conditions (functional and cognitive impairments and falls) have been documented (Brown et al. 2012), as have increased morbidity and mortality from cardiovascular diseases linked with behavioural risk factors such as illicit drug and/or tobacco use, and poor access to early care (Jones et al. 2009). Homeless individuals have higher rates of hospital admission and longer stays once admitted (Hwang et al. 2013) but are less likely to engage with preventive health services or adhere to medication (Fazel et al. 2014). These factors contribute to premature mortality. In Dublin, standardized mortality ratios (SMRs) were 3–10 times higher in homeless men and 6–10 times higher in homeless women compared with the general population between 2011 and 2015, with overdose related to substance use the leading cause of death (Ivers et al. 2019).

In Ireland, because there is currently no mechanism for linking homelessness and health outcomes data, understanding housing-related health disparities requires synthesizing empirical evidence from individual studies. To date, only one review of literature has been conducted relating to homelessness and health in the Republic of Ireland. McNeill et al. (2022) reviewed access to healthcare for people experiencing homelessness in the UK and Ireland (McNeill et al. 2022), identifying a need for improved staff education, flexibility of systems, service coordination, patient preparedness and holistic care across the two countries. Also in 2022, Health Service Executive Ireland (HSE) documented a need for improved supports across healthcare services for homeless people who experience chronic and complex health conditions, especially those persistently homeless, sleeping rough, or with substance use disorders (Health Services Executive Ireland 2022). Meeting and monitoring these needs first requires understanding baseline differences in health and healthcare outcomes; a task of particular urgency in light of Ireland’s ongoing housing crisis and sustained increases in homelessness (Department of Housing, Local Government and Heritage 2022), evidence that disparities in mortality between homeless and housed people are worsening over time (Ivers et al. 2019), and the recent entrenchment of said disparities during the COVID-19 pandemic (Corey et al. 2022).

To facilitate discussion regarding directions for targeted research and interventions, the two primary aims of this study are to provide an overview of research on the relationship between homelessness and health in the Republic of Ireland, and to synthesize the existing evidence on housing-related disparities in health status, and health care access and use. We include both objectives because – in addition to the identification of health disparities – the review will identify the extent to which research exists to address them.

## Methods

### Search strategy

This systematic review was registered to PROSPERO (ID: CRD42022348943) (Ingram and Perrotta 2022), conducted in accordance with Cochrane guidelines (Higgins et al. 2022), and reported according to the Preferred Reporting Items for Systematic reviews and Meta-Analyses (PRISMA) statement (Additional File 1) (Page et al. 2021). Articles were retrieved on 18 November 2022 through a keyword search in 11 bibliographic databases: PsychInfo, PsychArticles, Cochrane library, PubMed, Embase, CINAHL, Web of Science, Social Sciences (Full Text), Econlit, Scopus, and Academic Search Complete. Search terms related to **health** (health* OR risk OR disease OR mortality), **homelessness** (homeless* OR ‘insecure housing’ OR ‘unstable housing’ OR ‘precarious housing’), and the **Republic of Ireland** (Ireland* OR Irish). Searches were restricted to articles published since January 2012 to correspond with the dates for which official homelessness data exists in Ireland (Department of Housing, Local Government and Heritage 2022).

### Study selection

Identified citations were collated, uploaded into Rayyan (Ouzzani et al. 2016), and duplicates removed. Initial screening included any peer-reviewed article or conference abstract in English with empirical data on health status, or health care access or use, of individuals experiencing homelessness in Ireland. Under Ireland’s Housing Act 1988, homelessness is defined as (1) having no accommodation available that can reasonably be stayed in or living in a hospital, county home, night shelter or other such institution, and (2) being unable to provide accommodation from one’s own resources (Government of Ireland 1988). One researcher screened titles and abstracts and two researchers independently screened remaining full texts to exclude articles conducted outside of the Republic of Ireland, without an empirical component, or not related to homelessness and health. References from relevant articles and systematic reviews were reviewed for eligibility. The aim of this initial screen was to map existing qualitative and quantitative evidence on the health of individuals experiencing homelessness in Ireland.

In a second screening stage, two researchers independently re-examined the full texts of included articles to identify quantitative studies containing a method for comparing at least one health indicator between individuals experiencing homelessness and the general population in Ireland. As the measurement of health disparities requires accounting for group size (Penman-Aguilar et al. 2016), studies with incomplete denominators in the homeless and/or reference group were excluded. Complete study selection criteria are shown in Additional File 2. Disagreements that arose between reviewers at each stage of the selection process were resolved through discussion.

### Data extraction and analysis

Data from all studies meeting first-round inclusion criteria were extracted into a database in Excel describing study design and location, sample size, health topic(s) addressed, whether articles focused on health status or health care access, quality, or use, and additional keywords (Additional File 3). Reasons for inclusion/exclusion in further quantitative synthesis were marked as ‘include’, ‘qualitative’, ‘no comparison data’, or ‘missing denominator(s)’. A separate database was created for articles meeting second-round inclusion criteria in which the sampled population, study design, health indicator of interest, and method for measuring and analysing disparity were recorded (Additional File 4). Where possible, reviewers extracted two-by-two table contents for relevant indicators and calculated relative risk (RR), absolute risk reduction (ARR) and 95% confidence intervals (CI). Continuous measures of effect were recorded as mean and standard deviation (SD) in the homeless and non-homeless groups, or recorded as reported by study authors (e.g. SMR, incidence rate ratio (IRR), percent increase over time).

Studies with comparable outcome measures and study populations were included in meta-analyses. Pairwise random-effects meta-analyses were performed using the DerSimonian–Laird method to estimate the pooled RR of comparable health outcomes in the homeless vs. non-homeless population. Cochran’s Q test was performed to estimate whether total variation differed significantly from expected variation. τ^2^ (T2) and I^2^ statistics estimated between-study variance, with I^2^ > 75% indicating ‘high’ heterogeneity (Deeks et al. 2022). All data were analysed in R version 4.0.2 (Balduzzi et al. 2019).

### Quality assessment

Strength of study design was assessed using a quality checklist adapted from other systematic reviews (Harden et al. 2004; De Silva et al. 2005; Cook et al. 2013). Studies measuring health disparities were given a score from 0 to 5 based on the following criteria: (1) sample selected was something other than a convenience sample to reduce sample selection bias and allow for generalizability to a larger population; (2) clear description of methods used to collect, analyse data, and assess disparities; (3) attempt made to establish non-self-reported/objective assessment of the data; reliability and validity of the data; (4) sampled from multiple sites/communities/localities (rather than one site) to increase sample representativeness; and (5) sufficient sample size and standard errors accurately reported.

## Results

### Map of existing research on homelessness and health in Ireland

**Fig. 1:**
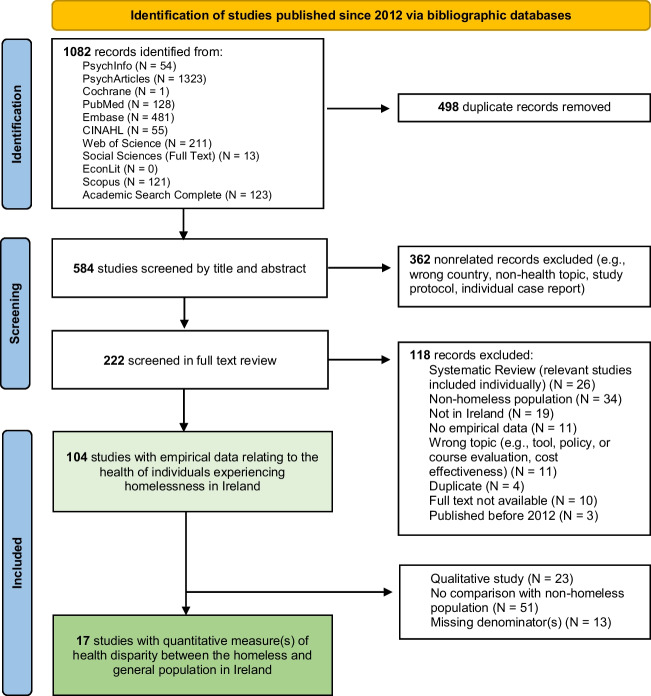
PRISMA flowchart

Of the 584 non-duplicate records published since 2012 identified via bibliographic databases, 104 contained empirical data on the health of homeless individuals residing in the Republic of Ireland (Fig. 1). These are listed and numbered M1 to M104 in Additional File 5.

One fifth of included records were conference abstracts (N = 20). Studies were primarily cross-sectional (N = 33), retrospective reviews (N = 20), or of qualitative design (N = 23). Ten studies employed mixed methods (e.g. quantitative surveys and interviews) [M1, M7, M13, M46, M51, M53, M68, M70, M72, M90]: nine studies designed, piloted, and assessed new interventions [M2, M18, M22, M25, M36, M50, M66, M77, M100]. Other study designs included prospective cohort studies [M6, M24, M37, M73], a case-control study [M76], epidemiological investigation [M75], follow-up study [M12], policy analysis [M19], and prospective clinical audit [M99]. The median number of studies published per year was 10 (min: 4 in 2014, max: 19 in 2019) with a noticeable jump in annual number of publications after 2016.

**Fig. 2:**
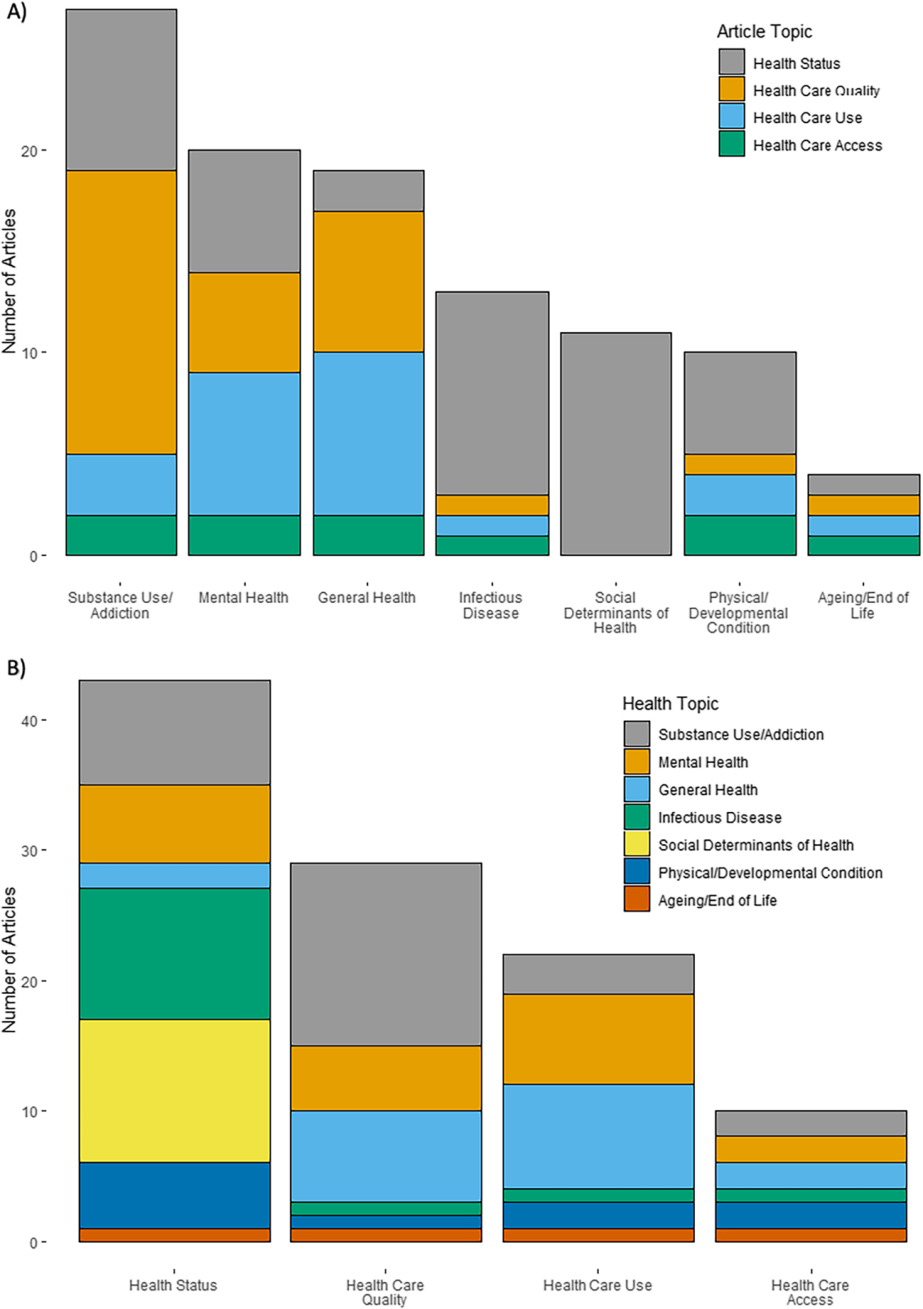
Quantity of Existing Research on the Health of Individuals Experiencing Homelessness in the Republic of Ireland by (A) Health Category, and (B) Article Topic: 2012 – 2022

Figure 2 maps the existing research by health topic, and article topic (i.e., focus on health status or health care access, quality, or use). In order of descending frequency, topics addressed included substance use and addiction (N = 27), mental health (N = 20), general health (N = 19), infectious disease (N = 13), social determinants of health (SDOH) (N = 11), physical and developmental conditions (N = 10), and ageing/end of life (N = 4).

Specific health topics addressed in each article are displayed in Table 1 and can be summarised as follows:

- **Substance Use/Addiction**: Studies reported rates of illicit drug overdose [M1–3], gambling [M4], relapse [M6], and sleep disturbances [M5] amongst homeless people who inject drugs (PWID). The quality of various harm reduction strategies was assessed [M17–21], specifically housing first vs. traditional staircase approaches [M9–16]. Singular studies reported on methadone use [M23], cervical screening uptake [M25], access to contraception [M26] and availability of addiction treatment programmes [M27].
- **Mental Health**: The prevalence and severity of mental illness amongst Ireland’s homeless population was reported generally [M28–30], and relating specifically to suicide, self-harm [M31, M32] and complex post-traumatic stress disorder (CPTSD) [M33]. Studies reported on factors associated with the quality of mental health services [M34], the effectiveness of mindfulness training and psychologically informed approaches [M35, M36], and discrepancies in medicines reconciliation for homeless individuals with mental illness [M38]. The frequency of homeless emergency department (ED) mental health presentations [M39, M40], hospital treated self-harm [M41, M42], and psychiatric inpatient care [M43–45] was reported, as were barriers to accessing mental health care [M46, M47].
- **General Health**: Studies that reported on the general health of homeless individuals looked into mortality rates [M48], perceived quality of life [M49], and how mobile health clinics, integrated care approaches [M50–52], and doctor–patient interactions [M53–55] affected homeless individuals’ experiences with health care. Studies reported on ED utilisation [M57–62], primary health care utilisation [M63, M64], and access to primary care [M65, M66].
- **Infectious Diseases**: Ten studies focused on hepatitis C (HCV), of which eight reported results from the HepCare Ireland HCV screening and linkage to care program targeting the homeless [M67–74]. A separate study looked at the success of telementoring as an HCV intervention [M77], and another at barriers to HCV treatment in PWID. Studies also looked at the prevalence [M75, M76] and use of HIV care among homeless PWID [M78].
- **SDOH**: Qualitative studies explored how lack of secure housing impacted child development, adolescent health, women’s health, family relationships and overall physical and mental health [M80–87]. Levels of social exclusion [M88] and access to nutritious food [M89, M90] were investigated.
- **Physical/Developmental Conditions**: Studies looked into rates of autism [M91], venous thromboembolism [M92], and diabetes [M94]; physical functioning [M93] and oral health [M95]; and access to/use of dermatology [M96], lung cancer [M97], epilepsy [M100] and rehabilitation services [M99].
- **Ageing/end of life**: Four studies assessed the availability of and satisfaction with palliative care services for homeless individuals in Ireland [M101–104].

**Table 1:** Map of Existing Research on the Health of Individuals Experiencing Homelessness in the Republic of Ireland: 2012–2022

	Health status	Health care quality	Health care use	Health care access
Substance use/addiction	• Drug Overdose [M1–3] • Gambling [M4] • Sleep [M5] • Drug Relapse [M6] • Factors Influencing health of PWID [M7, M8]	• Housing First vs. Traditional Staircase approach [M9–16] • Harm Reduction/OST [M17–21] • Smoking Intervention [M22]	• Methadone Use [M23] • Healthcare usage of PWID [M24] • Cervical Screening Uptake [M25]	• Contraception [M26] • Addiction Treatment [M27]
Mental health	• Prevalence/Severity of mental illness [M28–30] • Self-Harm [M31, M32] • CPTSD [M33]	• Factors associated with quality of services [M34] • Psychologically Informed Approaches [M35, M36] • Availability of social support [M37] • Medicines reconciliation [M38]	• ED mental health presentations [M39, M40] • Hospital-treated self-harm [M41, M42] • Psychiatric Inpatients Profile [M43–45]	• Access/barriers to mental health care [M46, M47]
General health	• Mortality [M48] • Perceived Quality of Life [M49]	• New approaches (mobile clinics, integrated care) [M50–52] • Doctor-patient interactions [M53–55] • Experience in hospital [M56]	• ED utilisation [M57–62] • Use of Health Services [M63, M64]	• Access/barriers to general health care [M65, M66]
Infectious disease	• HepCheck/HepCare: intensified HCV screening and linkage to care program [M67–74] • HIV in PWID [M75, M76]	• Telementoring HCV Intervention [M77]	• High-need users of acute, unscheduled HIV care [M78]	• Barriers to HCV treatment in PWID [M79]
Social determinants of health	• Interaction between social, built environment and health [M80–87] • Level of social exclusion [M88] • Nutrition [M89, M90]			
Physical/developmental condition	• Autism [M91] • Venous Thromboembolism (VTE) [M92] • Physical functioning [M93] • Diabetes [M94] • Oral Health [M95]		• Dermatology [M96] • Patient help-seeking for lung cancer [M97] • ED frequency for seizure [M98]	• Unmet rehabilitation needs [M99] • Epilepsy Care [M100]
Ageing/end of life	• Ear, nose, and throat cancer [M101]	• Satisfaction with palliative care [M102]	• Palliative care uptake in hostels [M103]	• Palliative care needs [M104]

The complete database of included studies is available as Additional File 3 along with descriptions of included variables.

### Health disparities amongst individuals experiencing homelessness

#### Study characteristics

The second-screening stage identified 14 peer-reviewed studies and three conference abstracts with indicators of health disparities published between 2014 and 2022 (Table 2). Study designs were primarily cross-sectional [M39, M45, M79, M80, M84, M88] or retrospective reviews of hospital data [M32, M41, M42, M48, M57, M58, M62, M92, M98]. One prospective cohort study [M37] and case control study [M76] were identified. Eleven studies took place in Dublin (capital city metropolitan area with a population of circa 1.25 million) [M37, M39, M48, M57, M58, M76, M79, M80, M84, M92, M98], one in Limerick (third largest city nationally with a population of circa 0.09 million) [M45], and one in Cork (second largest city nationally with a population of circa 0.22 million) [M62]. Four studies used national data [M32, M41, M42, M88]. Data were collected from hospital EDs [M39, M57, M58, M62, M80, M84] and psychiatric units [M37, M45], specialized epilepsy [M98] and fibro scanning services [M79], or public databases, including the National Registry of Deliberate Self-Harm Ireland [M32, M41] and Dublin Region Homeless Executive (DRHE) [M48]. One study requested clinician-provided data on HIV diagnoses [M76], and another – the only study conducted in a non-healthcare setting – surveyed homeless individuals linked with non-governmental organisations (NGO) [M88]. Studies lasted between 1 and 130 months (10 years).

**Table 2:** Characteristics of 17 studies containing a quantitative measure of health disparity between homeless vs. housed populations: Ireland 2012–2022

Author – Date [Ref]	Study design (Publication type)	Location	Setting	Health topic	Specific topic	Study dates	Study duration (months)	How was disparity measured?	Statistical analysis	Quality limitations^a^
Moloney et al. 2022 [M45]	Cross-Sectional (PR)	Limerick	Acute Psychiatric Unit (N=2)	Health Care Use	Psychiatric Care	NA	NA	Comparison of mental health/substance use characteristics between homeless vs housed psychiatric inpatients	Descriptive (N,%)	• Convenience sample • Self-report data • Small sample size
O’Brien et al. 2022 [M58]	Retrospective Review (PR)	Dublin	Temple St Children’s University Hospital ED	Health Care Use	ED Utilisation	2017-2020	47	Comparison of clinical details of ED presentation between non-homeless and homeless children under 16 years	Chi-squared or fisher exact test. Proportions presented as N (%) and 95% CI	• Convenience sample • Sampled from one site
O’Donnell et al. 2022 [M88]	Cross-Sectional (PR)	National	Participants recruited through NGOs in two Irish cities.	Social Determinant of Health	Social Exclusion	2019	4	Comparison of lived experiences between socially excluded (~80% homeless) and non-socially excluded participants	Descriptive (N,%)	• Convenience sample • Self-report data
Doran et al. 2021 [M98]	Retrospective Review (PR)	Dublin	St James Hospital Epilepsy Service	Health Care Use	Epilepsy/Seizure	2015-2019	59	Comparison of numbers of CT scan and radiation exposure between homeless vs housed cohorts of patients with seizure	Descriptive (N,%)	• Convenience sample • Sampled from one site
McLoughlin et al. 2021 [M39]	Cross-Sectional (PR)	Dublin South	St James Hospital ED	Health Care Use	ED Psychiatry Referrals	2019	1	Comparison of behaviours/psychiatric presentations between homeless vs. housed psychiatric referrals received through the ED	Descriptive (N,%)	• Convenience sample • Unclear methods • Sampled from one site • Small sample size
O’Brien et al. 2021 [M84]	Cross-Sectional (CA)	Dublin	Temple St Children’s University Hospital ED	Health Status	Child Health	2020-2021	2	Parental perceptions compared on the impact of housing situation on health and on use of primary and emergency healthcare services	Descriptive (N,%)	• Convenience sample • Unclear methods • Self-report data • Sampled from one site • Small sample size
Ewins et al. 2019 [M92]	Retrospective Review (CA)	Dublin	Hospital InPatient Enquiry data from Health Atlas Ireland	Health Status	Venous Thromboembolic Disease	2017	12	Comparison of number of emergency inpatient hospital admissions for those with any diagnosis of VTE between socially excluded individuals (including homeless) and general population	Descriptive (N,%)	• Unclear methods
Ui Bhroin, Kinahan, and Murphy 2019 [M62]	Retrospective Review (PR)	Cork	Mercy University Hospital ED	Health Care Use	Frequent ED Attendance	2016	12	Comparison of proportion of Frequent ED Attenders in homeless vs. non-homeless patients	Descriptive (N,%)	• Convenience sample • Sampled from one site
Arensman et al. 2018 [M41]	Retrospective Review (PR)	National	Self-harm presentations to hospital emergency departments (N=34) in Ireland (secondary analysis)	Health Care Use	Self-Harm	2004-2014	130	Comparison of care recommended following ED presentation for self-harm between homeless vs housed presenters	Descriptive (N,%)	
Hayes et al. 2019 [M80]	Cross-Sectional (CA)	Dublin	Temple St Children’s University Hospital ED	Health Status	Child Health	2011-2015	60	Parental perceptions compared on the impact of housing situation on health and on use of primary and emergency healthcare services	Descriptive (N,%)	• Convenience sample • Unclear methods • Self-report data • Sampled from one site • Small sample size
Ivers et al. 2019 [M48]	Retrospective Review (PR)	Dublin	Death data from cohort of homeless people in the Dublin region	Health Status	Mortality	2011-2015	60	Five-year SMR of homeless people living in the Dublin region compared to general population in Dublin	Standardized mortality ratios (SMR)	
Barrett et al. 2018 [M42]	Retrospective Review (PR)	National	Self-harm presentations to hospital emergency departments (N=34) in Ireland (secondary analysis)	Health Care Use	Self-Harm	2010-2014	60	Incidence rate ratio of homeless vs housed self-harm presentations and repeat presentations	Age-standardized incidence rate per 100,000 population	
Crowley et al. 2017 [M79]	Cross-Sectional (PR)	Dublin North	Fibro scanning clinic at National Drug Treatment Centre	Health Care Access	Hepatitis C Treatment	NA	NA	Comparison in proportion of study sample with Fibro scan score indicative of fibrosis	Descriptive (N,%)	• Convenience sample • Sampled from one site • Small sample size
Ní Cheallaigh et al. 2017 [M57]	Retrospective Review (PR)	Dublin	St James Hospital ED	Health Care Use	Unscheduled Hospital Care	2015	12	Comparison of clinical details of ED presentation between homeless and housed ED attenders	Descriptive (N,%)	• Sampled from one site
Renwick et al. 2017 [M37]	Prospective Cohort (PR)	Dublin/Mid-Leinster	In-patient and community admissions for first-episode psychosis	Social Determinant of Health	Social Support	2009-2012	37	Social network and support score (number of close friendships, quality of life, satisfaction) estimated based on living status (homeless vs. renting)	Estimates of Fixed Effects of Social Networks and Support Score	• Sampled from one site • Small sample size
Giese et al. 2015 [M76]	Case Control (PR)	Dublin	New HIV diagnoses notified by clinicians in Dublin	Health Status	HIV	2014-2015	24	Proportion of homeless participants in the case (HIV positive attendees of National Drug Treatment Centre) vs. control (HIV negative attendees of the National Drug Treatment Centre) groups	4x4 contingency table	• Sampled from one site • Small sample size
Larkin et al. 2014 [M32]	Retrospective Review (PR)	National	Records of self-harm based on emergency department case notes (Secondary Analysis)	Health Status	Self-Harm	2007-2011	60	Comparing proportion of homeless population vs. those in private residence experiencing 12-month repetition of self-harm presentations	Descriptive (N,%). Magnitudes of association demonstrated with Cramer's V.	

PR, peer-reviewed, CA, conference abstract, ED, emergency department, CT, computerised tomography

^a^Quality Checklist includes: (1) sample selected using something other than a convenience sample, (2) clear description of methods, (3) non-self-report/objective assessment, (4) sampled from multiple sites/communities/localities, (5) sufficient sample size

In terms of study quality, publications met between zero [M80, M84] and all five criteria [M32, M41, M42, M48] (Mean = Median = 3). The most common limitations were using a convenience sample and sampling from one site. Complete quality checklists are presented in Additional File 4.

**Fig. 3:**
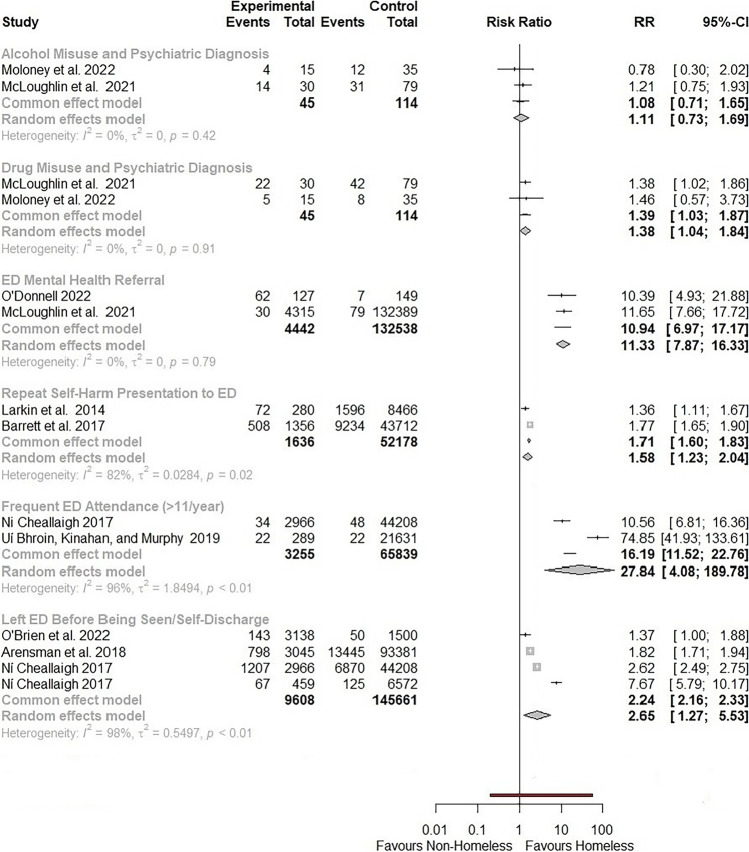
Forest plots of relative risks of comparable health outcomes amongst people experiencing vs. not experiencing homelessness in Ireland: 2012–2022

Health disparities reported were grouped into six categories: substance misuse, mental health and self-harm, health care use, health care access, physical health status, and social determinants of health. The researchers were able to extract two-by-two table contents and calculate RRs and ARRs for all but three studies that reported disparities as SMR [M48], age-standardized incidence rates [M42] and estimates of fixed effects [M37] (Table 3). Nine studies contained results that were amenable to meta-analyses of RRs, stratified by the following health outcomes: alcohol misuse amongst psychiatric inpatients [M39, M45], drug misuse amongst psychiatric inpatients [M39, M45], receiving an ED mental health referral [M39, M88], repeat self-harm presentation to ED [M32, M42], frequent (> 11) ED attendances per year [M57, M62], and leaving the ED before being seen/self-discharge [M41, M57, M58] (Fig. 3).

**Table 3:** Summary of reported health disparities between homeless vs. non-homeless populations: Ireland 2012–2022

Subject	Author – Date	Study population	N (Homeless)	N (Non-homeless)	Health disparity	Relative risk (95% CI)	Absolute risk reduction (95% CI)	Other measure
Alcohol Misuse/Dependence	Moloney et al. 2022	Psychiatric inpatients	15	35	Alcohol Misuse^a^	0.78 (0.30,2.02)	0.08 (–0.20,0.35)	
	McLoughlin et al. 2021	ED psychiatric referrals	30	79	Alcohol abuse/dependence^a^	1.21 (0.75,1.93)	–0.08 (–0.29,0.13)	
	O’Donnell et al. 2022	General population/non-healthcare setting	127	149	Has experienced period of problematic alcohol use	**5.79 (3.50,9.56)**	–0.48 (–58,–0.38)	
	O’Donnell et al. 2022	General population/non-healthcare setting	127	149	Has been involved in street drinking	**4.69 (2.92,7.55)**	–0.43 (–0.52,–0.32)	
Drug Misuse/Dependence	Moloney et al. 2022	Psychiatric inpatients	15	35	Substance Misuse^a^	1.46 (0.57,3.73)	–0.10 (–0.38,0.17)	
	McLoughlin et al. 2021	ED psychiatric referrals	30	79	Illicit drug abuse/dependence^a^	**1.38 (1.02,1.86)**	–0.2 (–0.40,–0.01)	
	O’Donnell et al. 2022	General population/non-healthcare setting	127	149	Has used ‘hard’ drugs	**7.33 (4.18,12.86)**	–0.51 (–0.61,–0.41)	
	O’Donnell et al. 2022	General population/non-healthcare setting	127	149	Has injected drugs	**51.62 (7.21,369.41)**	–0.34 (–0.42,–0.26)	
	Doran et al. 2021	ED attenders for seizure	88	88	Seizure related to substance abuse	**13 (1.74,97.26)**	–0.14 (–0.21,–0.06)	
Mental Health	Moloney et al. 2022	Psychiatric inpatients	15	35	Schizophrenia	1.48 (0.72,3.08)	–0.15 (–0.45,0.14)	
	Moloney et al. 2022	Psychiatric inpatients	15	35	Depression	0.58 (0.19,1.77)	0.14 (–0.11,0.40)	
	O’Donnell et al. 2022	General population/non-healthcare setting	127	149	Has been admitted to hospital for mental health issue^a^	**10.39 (4.93,21.89)**	–0.44 (–0.53,–0.35)	
	McLoughlin et al. 2021	General population/non-healthcare setting	4315	132389	ED psychiatric referral^a^	**11.65 (7.66,17.72)**	–0.006 (–0.009,–0.004)	
	McLoughlin et al. 2021	ED psychiatric referrals	30	79	Major mental illness (affective or psychotic disorder)	0.72 (0.35,1.49)	0.09 (–0.09,0.27)	
	O’Brien et al. 2021	Parents in triage at a tertiary paediatric ED	14	298	Housing negatively impacts child’s mental health (parent reported)	**4.88 (2.93,8.14)**	–0.49 (–0.75,–0.23)	
Self-Harm	McLoughlin et al. 2021	ED psychiatric referrals	30	79	Suicidal behaviour	0.86 (0.58,1.29)	0.08 (–0.13,0.29)	
	Arensman et al. 2018	Self-harm presentations to ED	3045	93381	Discharged from ED	**1.09 (1.04,1.13)**	–0.04 (–0.05,–0.02)	
	Arensman et al. 2018	Self-harm presentations to ED	3045	93381	General Admission	**0.48 (0.44,0.52)**	0.18 (0.16,0.19)	
	Arensman et al. 2018	Self-harm presentations to ED	3045	93381	Psychiatric Admission	**1.22 (1.11,1.33)**	–0.02 (–0.04,–0.01)	
	Barrett et al. 2018	General population/non-healthcare setting	2276	54848	Aged-standardized incidence rate ratio of self-harm ED presentations between 2010 and 2014 per 100,000 population			30
	Barrett et al. 2018	National self-harm presentations to ED	1356	43712	Repeat self-harm presentation to ED^a^	**1.77 (1.65,1.90)**	–0.16 (–0.19,–0.14)	
	Larkin et al. 2014	National self-harm presentations to ED	280	8466	Repeat self-harm presentation to ED^a^	**1.36 (1.11,1.67)**	–0.07 (–0.12,–0.02)	
Health Care Use	Ní Cheallaigh et al. 2017	General population/non-healthcare setting	1000	270000	ED attendance in 2015	**7.95 (7.78,8.13)**	–0.80 (–0.81,–0.78)	
	Ui Bhroin, Kinahan, and Murphy 2019	ED attenders	289	21631	Frequent ED attender (> 12 presentations/year)^a^	**74.85 (41.93,133.61)**	–0.08 (–0.11,–0.04)	
	Ní Cheallaigh et al. 2017	Presentations to ED	2966	44208	Frequent ED attender (> 11 presentations/ year)^a^	**10.56 (6.81,16.36)**	–0.01 (–0.01,–0.01)	
	O’Brien et al. 2022	ED attenders <16 years old	3138	1500	Increase in ED attendances between 2017 and 2019 (%)			74% vs 1%
	Ní Cheallaigh et al. 2017	Unscheduled hospital admissions	261	4853	Mean (SD) number of admissions over one year			2.79 (1.4) vs. 1.87 (1.1)
	Doran et al. 2021	ED attenders for seizure	88	88	Mean (SD) number of admissions over five years			11 (13.7) vs. 3.2 (3.5)
	Doran et al. 2021	ED attenders for seizure	88	88	Mean (SD) number of CT scans over five years			3 (4.8) vs. 1.2 (1.6)
	Doran et al. 2021	ED attenders for seizure	88	88	Mean (SD) radiation exposure in millisieverts (mSv) over five years			4.3 (6.8) vs. 1.7 (2.3)
	O’Brien et al. 2022	ED attenders <16 years old	3138	1500	Infant Presentation (Age < 12 months)	**1.58 (1.39,1.79)**	**–0.09 (–0.12,–0.07)**	
	Ní Cheallaigh et al. 2017	Unscheduled hospital admissions	261	4853	Bed days per capita of catchment population (days/person/annum)			4.4 vs. 0.3
	Ní Cheallaigh et al. 2017	Unscheduled hospital admissions	261	4853	ICU bed days per capita of catchment population (days/person/annum)			0.4 vs 0.03
	Ní Cheallaigh et al. 2017	Unscheduled hospital admissions	261	4853	Mean (SD) length of stay in days			9.41 (18.2) vs. 12.2 (24.9)
	Moloney et al. 2022	Psychiatric Inpatients	15	35	Voluntary Admission	0.75 (0.52,1.10)	0.22 (–0.04,0.48)	
	O’Brien et al. 2022	ED attenders <16 years old	3138	1500	Did not Wait to Be Admitted^a^	1.37 (0.99,1.88)	–0.01 (–0.02,–0.00)	
	Arensman et al. 2018	Self-harm presentations to ED	3045	93381	Left without being seen/Refused Admission^a^	**1.82 (1.71,1.94)**	–0.12 (–0.13,–0.10)	
	Ní Cheallaigh et al. 2017	Presentations to ED	2966	44208	Left before being seen/against medical advice^a^	**2.62 (2.50,2.75)**	–0.25 (–0.27,–0.23)	
	Ní Cheallaigh et al. 2017	Unscheduled hospital admissions	459	6572	Self-Discharged^a^	7.67 (5.79,10.12)	–0.13 (–0.16,–0.09)	
	Ní Cheallaigh et al. 2017	Unscheduled hospital admissions	459	6572	Discharged to home/homelessness	**0.90 (0.86,0.93)**	0.10 (0.13,10.43)	
Health Care Access	O’Brien et al. 2022	ED attenders <16 years old	3138	1500	Has GP	**0.73 (0.71,0.75)**	**0.26 (0.24,0.28)**	
	O’Brien et al. 2022	ED attenders <16 years old	3138	1500	Medical Card	**1.59 (1.45,1.73)**	**–0.17 (–0.20,–0.14)**	
	O’Brien et al. 2022	ED attenders <16 years old	3138	1500	Complete Vaccination	**0.88 (0.86,0.91)**	**0.09 (0.07,0.12)**	
Physical Health Status	Crowley et al. 2017	Opioid dependent patients living in North inner-city Dublin	37	22	Fibro scan score indicative of fibrosis (> 8.5 kilopascals (kPa))	0.86 (0.44,1.67)	0.06 (–0.20,0.31)	
	Crowley et al. 2017	Opioid dependent patients living in North inner-city Dublin	37	22	Fibro scan score indicative of liver disease (> 12.5kPa)	1.78 (0.54,5.90)	–0.12 (0.09,–9.36)	
	Giese et al. 2015	People Who Inject Drugs (PWID) in Dublin	32	21	HIV positive	0.66 (0.27,1.60)	0.11 (–0.13,0.36)	
	O’Brien et al. 2021	Parents in triage at a tertiary paediatric ED	14	298	Housing negatively impacts child’s physical health (parent reported)	**3.89 (2.02, 7.47)**	–0.34 (–0.60,–0.07)	
	Ewins et al. 2019	General population/non-healthcare setting	25500	4509167	Hospitalisation due to venous thromboembolism (VTE)	**10 (8.92,11.21)**	–0.01 (–0.01,–0.01)	
	Ivers et al. 2019	General population/non-healthcare setting	5481	991563	5-year standardised mortality ratio			6
Social Determinants of Health	O’Brien et al. 2022	ED attenders <16 years old	1975	876	Irish Ethnicity	**0.50 (0.47,0.54)**	**0.37 (0.34,0.41)**	
	O’Donnell et al. 2022	General population/non-healthcare setting	127	149	Was in state care as a child	**8.21 (3.32,20.34)**	–0.24 (–0.32,–0.16)	
	O’Brien et al. 2021	Parents in triage at a tertiary paediatric ED	14	298	Housing negatively impacts child’s social development (parent reported)	**5.06 (3.28, 7.82)**	–0.57 (–0.81,–0.33)	
	Hayes et al. 2019	Parents in triage at a tertiary paediatric ED	122	98	Fast-food meals twice or more per week (parent reported)	1.57 (0.70,3.53)	–0.10 (–0.30,0.10)	
	Hayes et al. 2019	Parents in triage at a tertiary paediatric ED	122	98	Limited ability to make and maintain friends (parent reported)	2.55 (0.82,7.94)	–0.11 (–0.30,0.06)	
	Hayes et al. 2019	Parents in triage at a tertiary paediatric ED	122	98	Limited ability to exercise and play (parent reported)	**3.00 (1.38,6.38)**	–0.24 (–0.45,–0.03)	
	Renwick et al. 2017	In-patient and community admissions for first-episode psychosis	5	217	Mean (SD) social network and support score (range: –3 to 3)			–2.34 (0.87) vs. –0.36 (0.65)

Bold entries indicate statistical significance (*p* < 0.05)

^a^Indicator included in meta-analysis (Fig. 3)

#### Substance misuse

Homelessness, when compared to the general population, was associated with a significantly higher risk of having misused alcohol in one’s lifetime (N = 276; RR 5.8 [95% CI 3.5, 9.6]; ARR –0.5 [95% CI –0.6, –0.4]) [M88]. However, pooled random effects meta-analysis demonstrated no significant differences in alcohol misuse between homeless and housed psychiatric inpatients (N = 159; pooled RR 1.11 [95% CI 0.7, 1.7]; Fig. 3). Homelessness was significantly associated with history of illicit drug use in both a non-healthcare setting (N = 276; RR 7.33 [95% CI 4.2, 12.9]; ARR –0.5 [95% CI –0.6, –0.4]) [M88] and amongst psychiatric inpatients (N = 159; pooled RR 1.38 [95% CI 1.04, 1.84]). Doran et al. (2021) found that of those presenting to ED for seizure, homeless individuals were more likely to experience seizure related to substance abuse (N = 176; RR 13.0 [95% CI 1.7, 97.3]; ARR –0.1 [95% CI –0.2, –0.06]) [M98].

#### Mental health and self-harm

Reported disparities in mental health outcomes varied. Pooled effects estimates revealed significantly higher risk of psychiatric admission amongst individuals experiencing homelessness (N = 136,980; pooled RR 11.3 [95% CI 7.9, 16.3]; Fig. 3). Amongst those having received a psychiatric referral, homelessness was not significantly associated with higher risk of schizophrenia, depression (N = 50) [M45] or affective or psychotic disorder (N = 109) [M39]. O’Brien et al. (2021) found homeless parents in triage at a paediatric ED at higher risk of indicating that their housing circumstances negatively impacted their child’s mental health (N = 312; RR 4.9 [95% CI 2.9, 8.1]; ARR –0.5 [95% CI –0.6, –0.2]).

Four studies assessed disparities in self-harm behaviours and related care [M32, M39, M41, M42]. Barrett et al. (2018) found the age-standardized incidence rate of self-harm ED presentations between 2010 and 2014 to be 30 times higher in Ireland’s homeless population than in the general population (N = 57,124). Of ED presentations for self-harm (N = 96,426), homeless presentations were at higher risk of discharge (RR 1.1 [95% CI 1.0, 1.13]; ARR –0.04 [95% CI –0.05, –0.02]) or psychiatric admission (RR 1.2 [95% CI 1.1, 1.3]; ARR –0.02 [95% CI –0.04, –0.01], and lower risk of general admission (RR 0.5 [95% CI 0.4, 0.52]; ARR 0.18 [95% CI 0.16,0.19]) [M41]. Homeless ED psychiatric referrals were not significantly more at risk of suicidal behaviour (N = 109; RR 0.9 [95% CI 0.6, 1.3]) [M39]. However, generally, homelessness was associated with risk of repeat self-harm presentation to ED (N = 53,814; pooled RR 1.6 [95% CI 1.2, 2.0]).

#### Health care use

Seven studies used hospital electronic patient records to investigate the relationship between homelessness and emergency care. Homeless individuals were eight times more likely to attend one Dublin hospital ED in 2015 (N = 271,000; RR 8.0 [95% CI 7.8, 8.1]; ARR –0.8 [95% CI –0.81, –0.78]) [M57]. They were also more likely to attend the ED multiple times. The relative risk of frequent ED attendance (> 11 visits/year) ranged from 10 to 75 (N = 69,094; pooled RR 27.8 [95% CI 4.1, 189.8]), with an estimated 1 to 8% of frequent ED presentations resulting from homelessness (Table 3) [M57, M62]. Between 2017 and 2019, O’Brien et al. (2022) calculated a 74% increase in homeless presentations to a Dublin paediatric ED compared to a 1% increase in housed presentations (N = 4638) [M57]. The same study found homelessness associated with higher risk of infant presentation (< 12 months) (RR 1.6 [95% CI 1.4,1.8]; ARR –0.09 [95% CI –0.1,–0.07]). Doran et al. (2021) looked at Dublin ED attendances for seizure over five years and found average attendances (11 ±13.7 vs 3.2 ±3.5) and number of CT scans (3 ±4.8 vs. 1.7 ±2.3) three times more likely in the homeless cohort, resulting in higher rates of radiation exposure (N = 176) [M98].

Once admitted to hospital, homeless patients had shorter hospital stays on average (9.41 ±18.2 vs. 12.2 ±24.9) but occupied 15 times more bed days per capita of the catchment population (4.4 vs. 0.3 days/person/annum) (N = 5114) [M57]. Across studies, homelessness was associated with a higher risk of leaving the ED before being seen or of self-discharge once admitted (N = 155,269; pooled RR 2.65 [95% CI 1.27, 5.53]; Fig. 3).

For ED attenders younger than 16, homelessness was associated with lower risk of presenting for injury or ‘being well’, and higher risk of presenting for respiratory viral, wheezing or gastro (N = 4638) [M58]. Homeless adult attenders were less likely to present for abdominal pain, chest pain or shortness of breath, and more likely to present for head injury or collapse (N = 47,062) [M57]. Upon admission, homelessness was positively associated with diagnoses of pneumonia/bronchitis, cellulitis, and seizure (N = 7031; see Additional File 6 for specific effect measures) [M57].

#### Health care access

Only one study reported disparities in healthcare access (N = 4638) [M58]. Homeless children attending Dublin paediatric ED were less likely to have a GP (RR 0.73 [CI 95% 0.71, 0.75]; ARR 0.26 [CI 95% 0.24, 0.28]) and to be completely vaccinated (RR 0.88 [CI 95% 0.86,0.91]; ARR 0.09 [CI 95% 0.07, 0.12]), but more likely to have a medical card (RR 1.6 [CI 95% 1.5, 1.7]; ARR –0.17 [CI –0.2,–0.14]).

#### Physical health status

Studies conducted by Crowley et al. (2017) and Giese et al. (2015) in cohorts of PWID did not reveal significant differences in the prevalence of fibrosis, liver disease (N = 59), nor HIV positivity (N = 53) according to housing status [M76, M79]. Homeless parents surveyed in ED triage were more likely to report that their housing situation negatively impacted their child’s physical health (N = 312; RR 3.9 [95% CI 2.0, 7.5]; ARR –0.34 [95% CI –0.6, –0.07]) [M84]. Individuals experiencing homelessness were at greater risk of hospitalisation due to VTE than the adult general population (N = 4,534,667; RR 10 [95% CI 8.9, 11.2]; ARR –0.01 [95% CI –0.01, –0.01]) [M92]. Between 2011 and 2015, five-year SMR were six times higher in homeless vs housed people living in the Dublin region (N = 997,044), with drugs and alcohol the leading cause of death (38%) [M48].

#### Social determinants of health

Concerning non-medical factors that influence health outcomes, three studies reported disparities in early childhood development [M80, M84, M88] and two relating to social inclusion [M37, M58]. Homeless parents surveyed in paediatric hospital triage were more likely to report negative impacts of housing on their child’s social development (N = 321; RR 5.1 [MCI 95% 3.3, 7.8]; ARR –0.6 [95% CI –0.8, –0.3]) [M84] and ability to exercise and play (N = 220; RR 3.0 [95% CI 1.4, 6.4]; ARR –0.2 [95% CI –0.5, –0.03]) [M80]. Alternatively, no significant differences in the frequency of fast-food meal consumption or ability to make and maintain friends were recorded (N = 220) [M80]. Homeless adults were more likely to have spent time in state care as a child (N = 276; RR 8.2 [95% CI 3.2, 20.3]; ARR –0.2 [95% CI –0.3, –0.2]) [M88] and reported fewer close friendships in adulthood (N = 222) [M37]. Children in one paediatric Dublin ED were less likely to be of Irish ethnicity (N = 2851; RR 0.5 [95% CI 0.47, 0.57]; ARR 0.37 [95% CI 0.34, 0.41) [M58].

## Discussion

### Study scope and quality

Our initial overview of research on homelessness and health in Ireland identified 104 studies, nearly half of which focused on substance use, addiction, and mental health. Of these, 17 studies reported disparities in health or healthcare-related outcomes, revealing associations between homelessness and substance misuse, self-harm presentations to ED, psychiatric admission, reduced access to GP, frequent ED presentation, premature departure from hospital, and increased risk of hospitalisation due to VTE, pneumonia/bronchitis, cellulitis, and seizure. That half of studies reporting health disparities were retrospective reviews is not surprising given the availability of hospital admissions data. Population-based health data such as disease registers can be difficult to access and/or to link with homelessness data due to lack of a unique health identifier (Moran 2016). As a result, we found that researchers outside of the acute care setting relied on surveys to assess differences in health, potentially biasing results due to convenience sampling, sample size, or self-report data. These limitations are not unique to Ireland. An international umbrella review of studies investigating associations between homelessness and any health outcome found only two meeting criteria for ‘convincing evidence’ as of 2021 (Fornaro et al. 2022). Authors found – as did we – a tendency for small sample sizes and high heterogeneity between studies.

Without comprehensive, accessible data, our review reveals a currently incomplete understanding of housing-related health disparities in Ireland. Moving forward, the usage of unique identifiers and linkage of health and social care data should be prioritized, as should the routine recording of housing status in primary care, hospital datasets, and national disease registries. Examples of successful linkages of homelessness and health datasets exist from Scotland and the US (Waugh et al. 2018; Trick et al. 2021).

### Substance misuse

Findings from our review are in line with international evidence, pointing to the disproportionate risk of alcohol and drug misuse amongst homeless individuals compared to the general population (Fazel et al. 2014) [M48,M88]. As of 2015, disparities in SMR calculated by Ivers et al. (2019) were largely attributable to drug and alcohol use [M48]. Though the proportion of substance-use related homeless deaths in the US was comparable to Ireland that same year (~30%) it has since skyrocketed to 82% due to proliferation of synthetic opioids, underlining the increasing severity of this crisis internationally (Cawley et al. 2022). Encouragingly, we found a growing body of research from Ireland focused on improving the quality and availability of addiction care and supports. Studies demonstrate the success of the Housing First vs. Traditional Staircase approach [M9–16], of creating a continuum of care for service users across all addiction and homeless services [M18], and of lowering thresholds to methadone provision, benzodiazepine provision, and naloxone availability [M19]. Less encouraging are lags in subsequent policy change and implementation. Ireland’s first Supervised Injection Facility was approved in 2015, for example, but none are operative as of 2023 (Harm Reduction International 2022; Health Services Executive Ireland 2023). We identified one study examining barriers preventing the operationalisation of effective harm reduction strategies and how to overcome them [M19]. This remains an important direction for future research.

### Mental Health

The fact that the association between homelessness and substance use was reduced/attenuated in cohorts of psychiatric inpatients (Fig. 3) aligns with evidence that a relationship between mental illness and substance use exists independently from homelessness (Maremmani et al. 2017). This brings to light the issue of dual diagnosis. The Irish mental health care system currently delineates between general and addiction psychiatric services, preventing individuals with both a substance misuse and mental health condition from accessing general mental health care (O’Carroll 2020). Individuals with a dual diagnosis are at higher risk of suicide, undoubtedly contributing to the disproportionate and increasing risk of self-harm and repeat self-harm presentation to ED identified amongst homeless cohorts included in this review [M32, M42]. However, homeless individuals presenting for self-harm (despite vulnerability to comorbid risk factors and repeat presentation) more frequently leave the ED without being seen [M41]. With a national model of integrated service provision for individuals with a dual diagnosis under development (Health Service Executive Ireland 2022), our review reveals a need for evidence relating to appropriate mental health care and support for homeless individuals with co-occurring substance use and mental health conditions.

It is also of note that studies reporting disparities in mental health status looked at risk of ED psychiatric referral (Fig. 3) or differences in psychiatric inpatients according to housing status, but that mental health disparities were not assessed outside of the healthcare setting. Prinsloo et al. (2012) found that only 24% of residents surveyed in a Dublin homeless hostel with a mental disorder diagnosis were attending psychiatric or addiction services, indicating the potential for hospital/health care data to miss individuals less inclined or able to engage with the mental health care system [M28]. Globally, homeless individuals are at elevated risk of personality disorder, bipolar disorder, and post-traumatic stress disorder (Fazel et al. 2014) and approximately half suffer from depressive symptoms and/or psychosis (Ayano et al. 2021). This suggests the likely scope and severity of mental health inequalities in Ireland. However, because estimates differ substantially by study and by demographic factor, our review reveals a need for better quality measurements that identify inequity in mental health outcomes in order to target appropriate, early interventions. Critically, this too could contribute to the prevention of suicide and unintentional overdose.

### Health care use

The most frequently reported disparities are related to ED presentations and hospital admissions. Included studies reported significantly elevated risk of ED attendance and frequent ED attendance, of leaving the ED without being seen, and – if admitted – of longer hospital stays amongst homeless individuals. Similar disparities have been widely reported internationally (Fazel et al. 2014; Vohra et al. 2022). However, a systematic review of homelessness and ED use found that the rate ratio of homeless vs housed ED visits reported by Ní Cheallaigh et al. (2017) was more than five times higher than results from comparable studies conducted in the US (Vohra et al. 2022). One explanation might be that the Dublin study was conducted most recently [M57], and that rates of ED attendance are increasing faster in homeless populations both in Ireland and abroad (Vohra et al. 2022) [M58]. Regardless, consensus across Irish studies regarding disproportionate rates of frequent ED attendance and premature departure (Fig. 3) demonstrate a problematic overreliance on acute care in a population with access to free primary and secondary healthcare [M63].

Studies in our evidence map offer some explanation. People experiencing homelessness have recurrent interactions with health professionals that leave them feeling excluded, discouraged, and more inclined to self-exclude from primary care [M54]. Once in hospital, homeless patients have reported lack of mental stimulation, insufficient detoxification programmes, poor communication by healthcare professionals, perceived discrimination associated with addiction, and lack of mental health supports as barriers to remaining in care [M55-56]. Dual diagnosis and substance use disorder are associated with after-hours visits to ED (Stenius-Ayoade et al. 2017), wait times can be long, and homeless patients may exhibit challenging behaviour that prevents staff from getting to examine underlying causes of repeat presentation [M54,M62]. Despite this evidence – and despite national recommendations for the identification of potential frequent attenders and development of patient-specific care plans as early as 2012 – our review confirms that stark disparities persist in the use of emergency care as of 2022 [M58].

### Physical health

That only a small number of studies (N = 5) reported disparities in physical health reveals – as with mental health outcomes – a need for better quality measurements. Studies revealed the disproportionate risk of diagnoses associated with injecting drug use [M57, M92]. Nationally, risk of hospitalisation due to VTE was 10 times higher in homeless individuals compared to the general population in 2019 [M92]. Beyond mortality risk, this is of particular consequence as Carroll et al. (2021) reported shortages in availability of rehabilitation services in Ireland to treat mobility issues that may be brought on by VTE [M99]. That the risk of seizure diagnosis was four times greater in homeless individuals may be indicative of increased rates of traumatic brain injury [M57,M98]. Internationally, homelessness is associated with disproportionate rates of chronic liver disease, HIV, HCV, and tuberculosis (TB) (Fazel et al. 2014; Hashim et al. 2022). However, when controlled for intravenous drug use, studies included in our review revealed no significant differences in HIV positivity or risk of liver disease according to housing status [M76,M79]. We identified a significant body of literature on ‘HepCare Ireland’, a project that has been improving national HCV outcomes through multiple-level testing and treatment interventions since 2016 [M70], but did not find information on TB. The prevalence of diabetes is comparable to general population estimates (8%) [M94]. Whereas cardiovascular diagnoses in hospital are less common [M57] despite evidence from Europe and Canada that homeless individuals have high rates of morbidity and mortality from cardiovascular disease (Fazel et al. 2014). Ní Cheallaigh et al. (2017) report increased risk of pneumonia/bronchitis diagnosis in hospital, but no differences in diagnoses of COPD or asthma [M57]. Rather than demonstrating baseline risk, disparities measured in an acute care setting may represent the fact that chronic conditions are more likely to be poorly controlled in homeless populations. Outside of the acute care setting, common physical health problems in homeless individuals include skin, dental, eye, joint, and asthma [M63, M95-96]. These rates have not been compared to the general population.

### Social Determinants of Health

Studies included in this review demonstrate higher rates of social exclusion amongst homeless cohorts [M37, M80, M84]. In Ireland as abroad, lack of housing reportedly negatively impacts child social development and levels of social support in adulthood, both factors that impact health and are aggravated by health status (Hwang et al. 2009) [M37,M84]. While not the aim of this review, it is imperative to recognize how SDOH lead to homelessness. Myriad factors – unemployment and poverty, migration, ageing, health or relationship difficulties, lack of affordable housing, and/or inadequate support when leaving care facilities or public institutions – can drive someone into precarious housing. In Ireland, for example, young people are discharged from care at age 18 and face sudden withdrawal of supports (Mayock et al. 2013). Many have developed drug and alcohol dependencies by this time and may struggle to find permanent housing [M81], beginning to explain the relationship between being in state care as a child and homelessness identified in this review [M88]. Encouragingly, we found a number of studies addressing homelessness as a combined medical and social issue in Ireland [M80-87]. One promising result is a national shift towards Housing First models of care [M9-16]. As well, research and practice can benefit from considering the health enabling traits related to homelessness such as high resiliency and survival skills (Lee et al. 2011).

Further exploration of the finding that homelessness is associated with non-Irish ethnicity in child ED presentation reveals overrepresentation of Roma, Black, and – to a lesser extent – Irish Traveller presentations in the homeless cohort [M58]. This is in stark contrast with demographics of the general homeless population which is primarily White Irish (Central Statistics Office 2016). In 2018, a Roma national needs assessment identified barriers to accessing social and medical services due to lack of documentation to prove residency, language barriers, not knowing about services or how to access them, and experiences of discrimination (Pavee Point and Department of Justice and Equality 2018). These findings, coupled with disproportionate rates of ED attendance, reveal a manifest need to better understand the health care needs and patterns of use specific to homeless members of ethnic minority communities. Even more so because there is limited national data collection of ethnicity, and ethnic/birth information is often missing from electronic medical records (Hannigan et al. 2019)[M58]. Our review identified no studies focused on the intersectionality of ethnicity, homelessness, and health in Ireland.

### Limitations & Strengths

It is important to note the limitations inherent to the exclusion of grey literature from this review. HSE Social Inclusion, Regional Homeless Executives, and non-profit organisations such as Simon Communities, Focus Ireland, the Ana Liffey Drug Project, and Partnership for Health Equity are leading the charge in homeless health equity and regularly publish reports on the state of housing and health in Ireland that complement findings from peer-reviewed literature. Another limitation is that this review did not report disparities in health according to age, gender, length of homelessness, or family status (single vs family homelessness); four factors known to significantly influence health outcomes (Ivers et al. 2019; O’Carroll 2020). Readers should be aware that the disparities reported apply predominately to homeless individuals linked with healthcare and may underestimate the severity of health difficulties facing those who are not. Finally, only a small number of studies could be included in meta-analyses due to variability of outcome variables and study populations. This resulted in small pools of comparable health outcomes (≤ 3) due to which meta-analytic results should be interpreted with caution.

Despite these limitations, this systematic review is an important contribution to the field as it provides the first map of evidence relating to homelessness and health in Ireland that can be used to inform and monitor future trends in research. By synthesizing existing evidence on housing-related health disparities, the review can facilitate cross-country and temporal comparisons in health outcomes and help guide the allocation of resources. Importantly, we have created two tools that can accelerate future searches for evidence. The first is a database of all relevant peer-reviewed studies and conference abstracts published since 2012 on homelessness and health in Ireland, indexed by Health Topic (Additional File 3). The second is a database containing all published, quantitative measures of homeless health disparities (Additional File 4).

## Conclusion

This review provides the first overview of research on homelessness and health conducted in Ireland, revealing a growing body of evidence on substance use, mental health status and access to/quality of related care. Studies reporting health disparities reveal associations between homelessness and substance misuse, self-harm presentations to ED, psychiatric admission, reduced access to GP, frequent ED presentation, premature departure from hospital, and increased risk of hospitalisation due to VTE, pneumonia/bronchitis, cellulitis, and seizure. While lack of routinely collected, accessible data prevents a complete understanding of Ireland’s status vis-à-vis homelessness and health equity, review findings shed light on an urgent need for more extensive harm reduction programmes, more appropriate mental health supports, and improved strategies for the identification and care of frequent ED attenders. Moving forward, national homelessness and health datasets should be expanded, linked, audited, and made more accessible. This will facilitate analysis of under-examined disparities in health relating to comorbid conditions, chronic and infectious diseases, and how ethnicity and SDOH interact with homelessness and health.

## Supplementary information

ESM 1 (DOC 68 kb)

ESM 2 (DOCX 18 kb)

ESM 3 (XLSX 47 kb)

ESM 4 (XLSX 74 kb)

ESM 5 (DOCX 28 kb)

ESM 6 (DOCX 15 kb)
