# Supplementary material for: Homelessness and health-related outcomes in the Republic of Ireland: a systematic review, meta-analysis and evidence map
Source: Z Gesundh Wiss. 2023 Jun 1:1–22. Online ahead of print. doi: 10.1007/s10389-023-01934-0 (PMC10233198; doi:10.1007/s10389-023-01934-0)
Supplement: Supplementary file 2 — (DOCX 18 kb) [file 10389_2023_1934_MOESM2_ESM.docx]

| Stage 1 Screening - Title & Abstract | | |
| --- | --- | --- |
|  | **Inclusion** | **Exclusion** |
| Population | - Homeless - Key informants reporting on needs of Homeless |  |
| Setting | Data collected in Republic of Ireland | - No data from the Republic of Ireland - Studies using international/European datasets that include data from Ireland but do not report outcomes specific to the RoI |
| Study design | Generates empirical primary or secondary data on a health topic:   - Quantitative studies - Qualitative studies | No empirical primary or secondary data on a health topic:   - Modelling studies - Commentaries/Letters - Individual case reports |
| Publication type | - Peer-reviewed publications - Conference Abstracts - Systematic reviews will be examined for individual studies meeting inclusion criteria | - Policy papers - Guidelines - Systematic reviews containing studies that meet inclusion criteria for the current review, hence are included individually - Grey literature (government documents and reports, pre-print articles, research reports, statistical reports) [***reason:*** *resources don’t allow for thorough grey literature search*] |
| Topic | Health status and/or health care access, quality, and utilisation:   - **Health conditions** (*e.g., addiction, diabetes, cancer, communicable/non-communicable disease, STI, pregnancy and childbirth, etc.)* - **Health behaviours**   (*e.g., nutrition, child development, tobacco use, vaccination, etc*.)   - **Health care access, utilisation, quality** - **Social determinants of health** *(e.g., social and community context, education, economic stability)* | - Animal study - Economic/ health care/housing policy not relating to health |
| Language | English | Any language that is not English |
| Date | Published in 2012 or later | Published before 2012 |
| At this stage in the Review Process, compiled records will include all of those with empirical (qualitative or quantitative) primary or secondary data on homeless health status and/or health care access, utilisation, and quality. | | |
| **Stage 2 – Full-text screening** | | |
|  | **Additional Inclusion** | **Additional Exclusion** |
|  | - Contains an empirical indicator of health status and/or access to care, quality of care in the general, housed population - Contains a method for comparing the health indicator(s) between the exposed and control groups (*e.g., relative risk, absolute difference, slope/relative index of inequality*) | - No empirical health indicators of the general housed population - No method for comparing health indicator(s) between the exposed and control groups (e.g., denominators missing) |
