# Supplementary material for: Homelessness and health-related outcomes in the Republic of Ireland: a systematic review, meta-analysis and evidence map
Source: Z Gesundh Wiss. 2023 Jun 1:1–22. Online ahead of print. doi: 10.1007/s10389-023-01934-0 (PMC10233198; doi:10.1007/s10389-023-01934-0)
Supplement: Supplementary file 5 — (DOCX 28 kb) [file 10389_2023_1934_MOESM5_ESM.docx]

**Scoping Review Studies by Health Topic**

Note: Articles in grey contain a quantitative measure of health disparity

**Substance Use/Addiction**

[M1] Barry T, Crowley D, Benton A, et al. Experience of drug overdose at an urban addiction clinic in Ireland. *Heroin Addiction and Related Clinical Problems* 2017; 19: 47–52.

[M2] Horan JA, Deasy C, Henry K, et al. Overdose risk perceptions and experience of overdose among heroin users in Cork, Ireland. Preliminary results from a pilot overdose prevention study. *Heroin Addiction and Related Clinical Problems* 2015; 17: 19–26.

[M3] Lynn TM, Lynn E, Keenan E, et al. Trends in Injector Deaths in Ireland, as Recorded by the National Drug-Related Deaths Index, M1998-2014. *J Stud Alcohol Drugs* 2018; 79: 286–292.

[M4] Condron I, Lyons S, Carew AM. Gambling in Ireland: profile of treatment episodes from a national treatment reporting system. *Ir J Psychol Med* 2022; 1–8.

[M5] Nkire N, Ekwegbalu UO, Iro C, et al. Subjective sleep complaints in patients attending a substance use disorder clinic. *Ir Med J* 2013; 106: 21–2.

[M6] Reddon H, Ivers J-H. Increased levels of hope are associated with slower rates of relapse following detoxification among people living with opioid dependence. *Addiction Research & Theory*. Epub ahead of print 2022. DOI: 10.1080/16066359.2022.2132238.

[M7] Van Hout MC, Bingham T. Open drug scenes and drug-related public nuisance: A visual rapid assessment research study in Dublin, Ireland. *Journal of Ethnicity in Substance Abuse* 2013; 12: 154–178.

[M8] Van Hout MC, Bingham T. ‘A costly turn on’: patterns of use and perceived consequences of mephedrone based head shop products amongst Irish injectors. *Int J Drug Policy* 2012; 23: 188–97.

[M9] Gaboardi M, Santinello M, Disperati F, et al. Working with People Experiencing Homelessness in Europe. *Human Service Organizations Management Leadership & Governance* 2022; 46: 324–345.

[M10] Greenwood RM, Manning RM. Mastery matters: consumer choice, psychiatric symptoms and problematic substance use among adults with histories of homelessness. *Health Soc Care Community* 2017; 25: 1050–1060.

[M11] Greenwood RM, Manning RM, O’Shaughnessy BR, et al. Homeless Adults’ Recovery Experiences in Housing First and Traditional Services Programs in Seven European Countries. *Am J Community Psychol* 2020; 65: 353–368.

[M12] Greenwood RM, Manning RM, O’Shaughnessy BR, et al. Structure and agency in capabilities‐enhancing homeless services: Housing first, housing quality and consumer choice. *Journal of Community & Applied Social Psychology* 2022; 32: 315–331.

[M13] Manning RM, Greenwood RM. Microsystems of Recovery in Homeless Services: The Influence of Service Provider Values on Service Users’ Recovery Experiences. *Am J Community Psychol* 2018; 61: 88–103.

[M14] Manning RM, Greenwood RM. Recovery in homelessness: The influence of choice and mastery on physical health, psychiatric symptoms, alcohol and drug use, and community integration. *Psychiatr Rehabil J* 2019; 42: 147–157.

[M15] Manning RM, Greenwood RM. Understanding Innovation in Homeless Service Provision: A Study of Frontline Providers’ Values-Readiness for Change. *Adm Policy Ment Health* 2019; 46: 649–659.

[M16] O’Shaughnessy B, Manning RM, Greenwood RM, et al. Home as a Base for a Well-Lived Life: Comparing the Capabilities of Homeless Service Users in Housing First and the Staircase of Transition in Europe. *Housing, Theory & Society* 2021; 38: 343–364.

[M17] Deacy JJP, Houghton F. The administration of naloxone: Social care worker perspectives and experiences. *Irish Medical Journal*; 112.

[M18] Kirby JE, Kitching A, Lane D, et al. Successful application of a cross-sectoral integrated care approach to addiction and homeless services - the experience from Southern Ireland. *International Journal of Integrated Care (IJIC)* 2017; 17: 1–2.

[M19] O’Carroll A, Duffin T, Collins J. Harm reduction in the time of COVID-19: Case study of homelessness and drug use in Dublin, Ireland. *Int J Drug Policy* 2021; 87: 102966.

[M20] Van Hout MC, Crowley D, McBride A, et al. Optimising treatment in opioid dependency in primary care: results from a national key stakeholder and expert focus group in Ireland. *BMC Fam Pract* 2018; 19: 103.

[M21] Horan JA, Van Hout MC. Mapping Service User Needs to inform a Supervised Injecting Room location in Cork, Ireland, EU. *Heroin Addiction and Related Clinical Problems* 2020; 22: 5–14.

[M22] Scheibein F, McGirr K, Morrison A, et al. An exploratory non-randomized study of a 3-month electronic nicotine delivery system (ENDS) intervention with people accessing a homeless supported temporary accommodation service (STA) in Ireland. *Harm Reduct J* 2020; 17: 73.

[M23] Glynn RW, Lynn E, Griffin E, et al. Self-Harm, Methadone Use and Drug-Related Deaths amongst Those Registered As Being of No Fixed Abode or Homeless in Ireland. *Ir Med J* 2017; 110: 631.

[M24] O’Connor G, McGinty T, Yeung SJ, et al. Cross-sectional study of the characteristics, healthcare usage, morbidity and mortality of injecting drug users attending an inner city emergency department. *Emerg Med J* 2014; 31: 625–9.

[M25] Haran M, Kelly JR, Kennedy L, et al. An audit of the cervical screening programme in the National Drug Treatment Centre (NDTC). *Ir J Med Sci* 2021; 190: 1379–1386.

[M26] Olioff J, O’Shea T, Naughton AM, et al. Assessing the need to provide contraceptive services to women attending addiction services at Cork-Kerry Community Healthcare. *BMC Proceedings*; 13. Epub ahead of print 2019. DOI: 10.1186/s12919-019-0167-8.

[M27] Welbel M, Matanov A, Moskalewicz J, et al. Addiction treatment in deprived urban areas in EU countries: Accessibility of care for people from socially marginalized groups. *Drugs: Education, Prevention & Policy* 2013; 20: 74–83.

**Mental Health**

[M28] Prinsloo B, Parr C, Fenton J. Mental illness among the homeless: prevalence study in a Dublin homeless hostel. *Ir J Psychol Med* 2012; 29: 22–26.

[M29] Springael M, Doherty A. The assessment of the severity of illness to the emergency department for psychiatric assessment. *BMC Proceedings*; 16. Epub ahead of print 2022. DOI: 10.1186/s12919-022-00235-w.

[M30] Hynes F, Kilbride K, Fenton J. A survey of mental disorder in the long-term, rough sleeping, homeless population of inner Dublin. *Ir J Psychol Med* 2019; 36: 19–22.

[M31] Dunne E, Duggan M, O’Mahony J. Mental health services for homeless: patient profile and factors associated with suicide and homicide. *Ir Med J* 2012; 105: 71–2, M74.

[M32] Larkin C, Corcoran P, Perry I, et al. Severity of hospital-treated self-cutting and risk of future self-harm: A national registry study. *Journal of Mental Health* 2014; 23: 115–119.

[M33] McQuillan K, Hyland P, Vallières F. Prevalence, correlates, and the mitigation of ICD-11 CPTSD among homeless adults: The role of self-compassion. *Child Abuse Negl* 2022; 127: 105569.

[M34] Costa D, Matanov A, Canavan R, et al. Factors associated with quality of services for marginalized groups with mental health problems in 14 European countries. *BMC Health Services Research*; 14. Epub ahead of print 3 February 2014. DOI: 10.1186/1472-6963-14-49.

[M35] Benson J, Brennan M. Keyworkers’ experiences and perceptions of using psychological approaches with people experiencing homelessness. *Housing Care and Support* 2018; 21: 51–63.

[M36] Maddock A, Hevey D, Eidenmueller K. Mindfulness Training as a Clinical Intervention with Homeless Adults: a Pilot Study. *International Journal of Mental Health and Addiction* 2017; 15: 529–544.

[M37] Renwick L, Owens L, Lyne J, et al. Predictors of change in social networks, support and satisfaction following a first episode psychosis: A cohort study. *Int J Nurs Stud* 2017; 76: 28–35.

[M38] Nikolić N. An explorative study on medicines reconciliation in the homeless with a mental illness. *Int J Pharm Pract* 2018; 26: 469–472.

[M39] McLoughlin A, Feeney A, Cooney J. Homelessness, emergency care and mental health. Inner-city emergency department psychiatry referrals: a retrospective descriptive analysis. *Ir J Med Sci* 2021; 190: 1201–1204.

[M40] McLoughlin C, McLoughlin A, Jain S, et al. The suburban-city divide: an evaluation of emergency department mental health presentations across two centres. *Ir J Med Sci* 2021; 190: 1523–1528.

[M41] Arensman E, Griffin E, Daly C, et al. Recommended next care following hospital-treated self-harm: Patterns and trends over time. *PLoS ONE* 2018; 13: 1–12.

[M42] Barrett P, Griffin E, Corcoran P, et al. Self-harm among the homeless population in Ireland: A national registry-based study of incidence and associated factors. *J Affect Disord* 2018; 229: 523–531.

[M43] Cowman J, Whitty P. Prevalence of housing needs among inpatients: a 1 year audit of housing needs in the acute mental health unit in Tallaght Hospital. *Ir J Psychol Med* 2016; 33: 159–164.

[M44] Daly A, Craig S, E OS. A Profile of Psychiatric In-Patient Admissions With No Fixed Abode (NFA) 2007-2016. *Ir Med J* 2019; 112: 853.

[M45] Moloney N, O’Donnell P, Elzain M, et al. Homelessness amongst psychiatric Inpatients: a cross-sectional study in the mid-west of Ireland. *Irish Journal of Medical Science* 2022; 191: 321–326.

[M46] Canavan R, Barry MM, Matanov A, et al. Service provision and barriers to care for homeless people with mental health problems across 14 European capital cities. *BMC Health Serv Res* 2012; 12: 222.

[M47] Priebe S, Matanov A, Barros H, et al. Mental health-care provision for marginalized groups across Europe: findings from the PROMO study. *European Journal of Public Health* 2013; 23: 97–103.

**General Health**

[M48] Ivers JH, Zgaga L, O’Donoghue-Hynes B, et al. Five-year standardised mortality ratios in a cohort of homeless people in Dublin. *BMJ Open* 2019; 9: e023010.

[M49] O’Brien KK, Schuttke A, Alhakeem A, et al. Health, perceived quality of life and health services use among homeless illicit drug users. *Drug and Alcohol Dependence* 2015; 154: 139–145.

[M50] Cheallaigh CN, Lawlee A-M, Sears J, et al. The Development of an Inclusion Health Integrated Care Programme for Homeless Adults in Dublin, Ireland. *International Journal of Integrated Care (IJIC)* 2018; 18: 1–2.

[M51] O’Carroll A, Irving N, O’Neill J, et al. A review of a GP registrar-run mobile health clinic for homeless people. *Ir J Med Sci* 2017; 186: 541–546.

[M52] Swabri J, Uzor C, Laird E, et al. Health status of the homeless in Dublin: does the mobile health clinic improve access to primary healthcare for its users? *Ir J Med Sci* 2019; 188: 545–554.

[M53] O’Reilly F, O’Carroll A. GP training for areas of deprivation and with marginalized groups: Does it make a difference? *European Journal of General Practice* 2017; 23: 147.

[M54] O’Carroll A, Wainwright D. Doctor-patient interactions that exclude patients experiencing homelessness from health services: an ethnographic exploration. *BJGP Open*; 5. Epub ahead of print June 2021. DOI: 10.3399/bjgpo.2021.0031.

[M55] Reynolds S, Burns E, Clinton C, et al. Attitudes, Skills and Knowledge (ASK): Patient-Identified Gaps inHealthcare Provider’s Approach to Homeless People. *Irish Journal of Medical Science* 2020; 189: S11.

[M56] O’Doherty L, Ni Cheallaigh C. Homeless patients’ experiences in hospital. *Irish Journal of Medical Science* 2017; 186: S211.

[M57] Ní Cheallaigh, Cullivan S, Sears J, et al. Usage of unscheduled hospital care by homeless individuals in Dublin, Ireland: a cross-sectional study. *BMJ Open* 2017; 7: e016420.

[M58] O’Brien, Quinn N, Joyce B, et al. Emergency department utilisation by homeless children in Dublin, Ireland: a retrospective review. *BMJ Paediatr Open*; 6. Epub ahead of print March 2022. DOI: 10.1136/bmjpo-2021-001368.

[M59] O’Farrell A, Evans DS, Allen M. The Epidemiology of Emergency In-Patient Hospitalisations Among Those with ‘No Fixed Abode’ (Homeless) 2005-2014: What Lessons Can Be Learnt. *Ir Med J* 2016; 109: 464.

[M60] Ramasubbu B, Donnelly A, Moughty A. Profile of frequent attenders to a Dublin inner city emergency department. *Irish Medical Journal* 2016; 109: 391.

[M61] Romero-Ortuno R, O’Riordan D, Silke B. Profiling the medical admissions of the homeless. *Acute Med* 2012; 11: 197–204.

[M62] Uí Bhroin S, Kinahan J, Murphy A. Profiling frequent attenders at an inner city emergency department. *Irish Journal of Medical Science* 2019; 188: 1013–1019.

[M63] Keogh C, O’Brien KK, Hoban A, et al. Health and use of health services of people who are homeless and at risk of homelessness who receive free primary health care in Dublin 58. *BMC Health Services Research*; 15. Epub ahead of print 2015. DOI: 10.1186/s12913-015-0716-4.

[M64] O’Carroll A, Wainwright D. Making sense of street chaos: an ethnographic exploration of homeless people’s health service utilization. *Int J Equity Health* 2019; 18: 113.

[M65] O’Donnell P, Tierney E, O’Carroll A, et al. Exploring levers and barriers to accessing primary care for marginalised groups and identifying their priorities for primary care provision: a participatory learning and action research study. *Int J Equity Health* 2016; 15: 197.

[M66] O’Reilly F, Doyle J, Keenan E. Innovative co-design of integrated services designed to improve access to health care. *International Journal of Integrated Care (IJIC)* 2017; 17: 1–2.

**Hep C/HIV**

[M67] Avramovic G, Oprea C, Surey J, et al. HepCare Europe—A service innovation project. HepCheck: Characteristics of the patient population with active infection as defined by HCV RNA. *International Journal of Infectious Diseases* 2020; 91: 246–251.

[M68] Lambert JS, Murphy C, Menezes DS, et al. Hepcheck Dublin: Homeless, Hep C & Competing Priorities. *International Journal of Integrated Care (IJIC)* 2017; 17: 1–2.

[M69] Barror S, Avramovic G, Oprea C, et al. HepCare Europe: a service innovation project. HepCheck: enhancing HCV identification and linkage to care for vulnerable populations through intensified outreach screening. A prospective multisite feasibility study. *J Antimicrob Chemother* 2019; 74: v39–v46.

[M70] Connolly SP, Avramovic G, Cullen W, et al. HepCare Ireland-a service innovation project. *Ir J Med Sci* 2021; 190: 587–595.

[M71] Glaspy S, Avramovic G, McHugh T, et al. Exploring and understanding HCV patient journeys- HEPCARE Europe project. *BMC Infect Dis* 2021; 21: 239.

[M72] Lambert JS, Murphy C, O’Carroll A, et al. The Dublin hepcheck study: Community based testing of HCV by point of care oraquick® HCV saliva test in homeless populations. *Journal of Hepatology* 2016; 64: S726.

[M73] Lambert JS, Murtagh R, Menezes D, et al. ‘HepCheck Dublin’: an intensified hepatitis C screening programme in a homeless population demonstrates the need for alternative models of care. *BMC Infect Dis* 2019; 19: 128.

[M74] Riogh ENA, Swan D, McCombe G, et al. Integrating hepatitis C care for at-risk groups (HepLink): baseline data from a multicentre feasibility study in primary and community care. *Journal of Antimicrobial Chemotherapy* 2019; 74: V31–V38.

[M75] Dunford L, Waters A, Neary M, et al. Role of phylogenetic analysis in epidemiological case definitions during an outbreak of HIV-1 in people who inject drugs in Ireland. *Virus Evolution* 2018; 4: S9–S10.

[M76] Giese C, Igoe D, Gibbons Z, et al. Injection of new psychoactive substance snow blow associated with recently acquired hiv infections among homeless people who inject drugs in dublin, ireland, M2015. *Eurosurveillance*; 20. Epub ahead of print 2015. DOI: 10.2807/1560-7917.ES.2015.20.40.30036.

[M77] Ní Cheallaigh C, O’Leary A, Keating S, et al. Telementoring with project ECHO: A pilot study in Europe. *BMJ Innovations* 2017; 3: 144–151.

[M78] Grant C, Bergin C, O’Connell S, et al. High-cost, high-need users of acute unscheduled HIV care: A cross-sectional study. *Open Forum Infectious Diseases*; 7. Epub ahead of print 2020. DOI: 10.1093/ofid/ofaa037.

[M79] Crowley D, Cullen W, Laird E, et al. Exploring Patient Characteristics and Barriers to Hepatitis C Treatment in Patients on Opioid Substitution Treatment Attending a Community Based Fibro-scanning Clinic. *J Transl Int Med* 2017; 5: 112–119.

**Social Determinants of Health**

[M80] Hayes AM, Joyce B, McNamara R, et al. Impact of Homelessness on Children Presenting to a Tertiary Emergency Department. *Archives of Disease in Childhood* 2019; 104: A262–A263.

[M81] Mayock P, Corr ML, O’Sullivan E. Moving on, not out: when young people remain homeless. *Journal of Youth Studies* 2013; 16: 441–459.

[M82] Mayock P, Sheridan S, Parker S. ‘It’s just like we’re going around in circles and going back to the same thing . . .’: The Dynamics of Women’s Unresolved Homelessness. *Housing Studies* 2015; 30: 877–900.

[M83] McCabe E, O’Connor J. Home remembered, relived and revised: A qualitative study exploring the experiences of home for homeless persons in supported accommodation. *European Journal of Psychotherapy & Counselling* 2016; 18: 290–303.

[M84] O’Brien, Quinn N, Joyce B, et al. Parental Perceptions Regarding the Impact of Housing on Child Health. *Ir Med J* 2021; 115: 652.

[M85] Nowicki M, Brickell K, Harris E. The hotelisation of the housing crisis: Experiences of family homelessness in Dublin hotels. *Geographical Journal* 2019; 185: 313–324.

[M86] Parker S, Mayock P. ‘They’re Always Complicated but That’s the Meaning of Family in My Eyes’: Homeless Youth Making Sense of ‘Family’ and Family Relationships. *Journal of Family Issues* 2019; 40: 540–570.

[M87] Taylor O, Loubiere S, Tinland A, et al. Lifetime, M5-year and past-year prevalence of homelessness in Europe: a cross-national survey in eight European nations. *BMJ Open* 2019; 9: e033237.

[M88] O’Donnell P, Hannigan A, Ibrahim N, et al. Developing a tool for the measurement of social exclusion in healthcare settings. *International Journal for Equity in Health*; 21. Epub ahead of print 15 March 2022. DOI: 10.1186/s12939-022-01636-1.

[M89] Ravikumar D, Kelly D. Dietary habits, food security and related health and wellbeing in the homeless population. *Obesity Reviews*; 21. Epub ahead of print 2020. DOI: 10.1111/obr.13118.

[M90] Share M. Housing, food and dignity: the food worlds of homeless families in emergency accommodation in Ireland. *Journal of Social Distress and the Homeless* 2020; 29: 137–150.

**Physical/Developmental Condition**

[M91] Boilson AM, Churchard A, Connolly M, et al. Screening for Autism Spectrum Condition Through Inner City Homeless Services in the Republic of Ireland. *J Autism Dev Disord* 2022; 1–12.

[M92] Ewins K, Ni Ainle F, Dunlea E, et al. Socially Excluded Persons in Ireland Have an Increased Annual Risk of Hospitalisation Due to Venous Thromboembolic Disease. *Blood* 2019; 134: 4702.

[M93] Kiernan S, C NC, Murphy N, et al. Markedly poor physical functioning status of people experiencing homelessness admitted to an acute hospital setting. *Sci Rep* 2021; 11: 9911.

[M94] Scott J, Gavin J, Egan AM, et al. The prevalence of diabetes, pre-diabetes and the metabolic syndrome in an Irish regional homeless population. *Qjm* 2013; 106: 547–53.

[M95] Van Hout MC, Hearne E. Oral health behaviours amongst homeless people attending rehabilitation services in Ireland. *J Ir Dent Assoc* 2014; 60: 144–9.

[M96] Awdeh F, O’Grady C, Gilhooley E, et al. Complexities surrounding the management of homeless patients in dermatology: An increasing phenomenon. *British Journal of Dermatology* 2019; 181: 192.

[M97] Saab MM, O’Driscoll M, FitzGerald S, et al. Primary healthcare professionals’ perspectives on patient help-seeking for lung cancer warning signs and symptoms: a qualitative study. *BMC Prim Care* 2022; 23: 119.

[M98] Doran EM, Stanila RM, Healy LA, et al. Computed tomography and emergency department frequency in homeless patients with seizures. *Seizure* 2021; 91: 72–74.

[M99] O’Carroll Á, O’Brien S, Harrington D, et al. The Unmet Rehabilitation Needs in an Inclusion Health Integrated Care Programme for Homeless Adults in Dublin, Ireland. *Int J Environ Res Public Health*; 18. Epub ahead of print 27 July 2021. DOI: 10.3390/ijerph18157917.

[M100] Doran E, Barron E, Healy L, et al. Improving access to epilepsy care for homeless patients in the Dublin Inner City: a collaborative quality improvement project joining hospital and community care. *BMJ Open Qual*; 10. Epub ahead of print April 2021. DOI: 10.1136/bmjoq-2021-001367.

**Ageing/End-of-Life**

[M101] Hudson A, O’Neill JP, McQuillan R. Exploring ENT cancer in homeless patients receiving palliative care: A case series. *Irish Journal of Medical Science* 2016; 185: S162.

[M102] Conneely A, Marshall S, Bristowe K, et al. Qualitative study exploring the experience of homelessness staff working with homeless people with life-limiting illnesses in Dublin, Ireland. *Palliative Medicine* 2018; 32: 88–89.

[M103] Hudson AE, McQuillan R. Providing palliative care in hostels: A case series. *Palliative Medicine* 2016; 30: NP51.

[M104] Conneely A, Ivers JH, Barry J, et al. Estimation of Palliative Care Needs of People Experiencing Homelessness Using Mortality Data and Cause-Of-Death. *BMJ Supportive and Palliative Care* 2021; 11: A43.
