# Supplementary material for: Homelessness and health-related outcomes in the Republic of Ireland: a systematic review, meta-analysis and evidence map
Source: Z Gesundh Wiss. 2023 Jun 1:1–22. Online ahead of print. doi: 10.1007/s10389-023-01934-0 (PMC10233198; doi:10.1007/s10389-023-01934-0)
Supplement: Supplementary file 6 — (DOCX 15 kb) [file 10389_2023_1934_MOESM6_ESM.docx]

**Additional Table.** Disparities in ED presenting complaints and hospital diagnoses: Ireland 2012-2022

| **Subject** | **Author - Date** | **Study Population** | **N** | **Health Disparity** | **Relative Risk (95% CI)** | **Absolute Risk Reduction (95% CI)** | **Other Measure** |
| --- | --- | --- | --- | --- | --- | --- | --- |
| ED Presentations | O'Brien et al. 2022 | ED attenders <16 years old | 4638 | Presenting for Injury | **0.63 (0.57,0.70)** | **0.12 (0.08,0.14)** |  |
| ED Presentations | O'Brien et al. 2022 | ED attenders <16 years old | 4638 | Presenting for Respiratory Viral | **1.63 (1.37,1.95)** | **-0.06 (-0.08,-0.04)** |  |
| ED Presentations | O'Brien et al. 2022 | ED attenders <16 years old | 4638 | Presenting for Other Viral | 0.99 (0.81,1.20) | 0.001 (-0.02,0.01) |  |
| ED Presentations | O'Brien et al. 2022 | ED attenders <16 years old | 4638 | Presenting for Infection | 1.05 (0.83,1.35) | -0.003 (-0.02,0.01) |  |
| ED Presentations | O'Brien et al. 2022 | ED attenders <16 years old | 4638 | Presenting for Wheezing | **1.80 (1.40,2.33)** | **-0.04 (-0.05,-0.02)** |  |
| ED Presentations | O'Brien et al. 2022 | ED attenders <16 years old | 4638 | Presenting for Gastro | **1.62 (1.26,2.06)** | **-0.03 (-0.05,-0.02)** |  |
| ED Presentations | O'Brien et al. 2022 | ED attenders <16 years old | 4638 | Presenting for Bacterial Respiratory Tract Infection | 1.22 (0.94,1.59) | -0.01 (-0.02,0.00) |  |
| ED Presentations | O'Brien et al. 2022 | ED attenders <16 years old | 4638 | Presenting for ‘Well Child’ | **0.76 (0.58,0.99)** | 0.01 (-0.00, 0.03) |  |
| ED Presentations | Ní Cheallaigh et al. 2017 | Presentations to ED | 47062 | Presenting for Limb Problems | 0.67 (0.61,0.74) | 0.06 (0.05,0.08) |  |
| ED Presentations | Ní Cheallaigh et al. 2017 | Presentations to ED | 47062 | Presenting for Abdominal Pain | **0.39 (0.33,0.47)** | 0.06 (0.05,0.07) |  |
| ED Presentations | Ní Cheallaigh et al. 2017 | Presentations to ED | 47062 | Presenting for Chest Pain | **0.38 (0.31,0.47)** | 0.05 (0.04,0.05) |  |
| ED Presentations | Ní Cheallaigh et al. 2017 | Presentations to ED | 47062 | Presenting for Unwell Adult | 0.97 (0.84,1.12) | 0.00 (-0.01,0.01) |  |
| ED Presentations | Ní Cheallaigh et al. 2017 | Presentations to ED | 47062 | Presenting for Shortness of Breath | **0.48 (0.39,0.59)** | 0.03 (0.03,0.04) |  |
| ED Presentations | Ní Cheallaigh et al. 2017 | Presentations to ED | 47062 | Presenting for Head Injury | **1.33 (1.14,1.55)** | -0.01 (-0.02,-0.01) |  |
| ED Presentations | Ní Cheallaigh et al. 2017 | Presentations to ED | 47062 | Presenting for Collapsed Adult | **1.42 (1.19,1.68)** | -0.01 (-0.02,-0.01) |  |
| ED Presentations | Ní Cheallaigh et al. 2017 | Unscheduled hospital admissions | 7031 | Diagnosed with acute exacerbation of COPD/asthma | 0.87 (0.58,1.30) | 0..01 (-0.01,0.03) |  |
| ED Presentations | Ní Cheallaigh et al. 2017 | Unscheduled hospital admissions | 7031 | Diagnosed with pneumonia/bronchitis | **1.97 (1.51,2.57)** | -0.06 (-0.09,-0.03) |  |
| ED Presentations | Ní Cheallaigh et al. 2017 | Unscheduled hospital admissions | 7031 | Diagnosed with syncope and collapse | 1.39 (0.94,2.05) | -0.02 (-0.04,0.01) |  |
| ED Presentations | Ní Cheallaigh et al. 2017 | Unscheduled hospital admissions | 7031 | Diagnosed with cellulitis | **2.07 (1.34,3.2)** | -0.03 (-0.05,-0.01) |  |
| ED Presentations | Ní Cheallaigh et al. 2017 | Unscheduled hospital admissions | 7031 | Diagnosed with seizure | **4.20 (2.98,5.92)** | -0.07 (-0.09,-0.04) |  |
